# A mobile app to capture EPA assessment data: Utilizing the consolidated framework for implementation research to identify enablers and barriers to engagement

**DOI:** 10.1007/s40037-020-00587-z

**Published:** 2020-06-05

**Authors:** John Q. Young, Rebekah Sugarman, Jessica Schwartz, Matthew McClure, Patricia S. O’Sullivan

**Affiliations:** 1 Donald and Barbara Zucker School of Medicine at Hofstra/Northwell and the Zucker Hillside Hospital at Northwell Health, Hempstead, NY USA; 2 Department of Medicine, University of California at San Francisco School of Medicine, San Francisco, USA

**Keywords:** Workplace-based assessment, Competency-based assessment, Entrustable professional activities, Mobile technology, App, Consolidated framework for implementation research, Implementation science, Psychiatry, Qualitative methods

## Abstract

**Introduction:**

Mobile apps that utilize the framework of entrustable professional activities (EPAs) to capture and deliver feedback are being implemented. If EPA apps are to be successfully incorporated into programmatic assessment, a better understanding of how they are experienced by the end-users will be necessary. The authors conducted a qualitative study using the Consolidated Framework for Implementation Research (CFIR) to identify enablers and barriers to engagement with an EPA app.

**Methods:**

Structured interviews of faculty and residents were conducted with an interview guide based on the CFIR. Transcripts were independently coded by two study authors using directed content analysis. Differences were resolved via consensus. The study team then organized codes into themes relevant to the domains of the CFIR.

**Results:**

Eight faculty and 10 residents chose to participate in the study. Both faculty and residents found the app easy to use and effective in facilitating feedback immediately after the observed patient encounter. Faculty appreciated how the EPA app forced brief, distilled feedback. Both faculty and residents expressed positive attitudes and perceived the app as aligned with the department’s philosophy. Barriers to engagement included faculty not understanding the EPA framework and scale, competing clinical demands, residents preferring more detailed feedback and both faculty and residents noting that the app’s feedback should be complemented by a tool that generates more systematic, nuanced, and comprehensive feedback. Residents rarely if ever returned to the feedback after initial receipt.

**Discussion:**

This study identified key enablers and barriers to engagement with the EPA app. The findings provide guidance for future research and implementation efforts focused on the use of mobile platforms to capture direct observation feedback.

**Electronic supplementary material:**

The online version of this article (10.1007/s40037-020-00587-z) contains supplementary material, which is available to authorized users.

## Introduction

The adoption of competency-based frameworks has highlighted the need for workplace-based assessment (i.e., “what doctors actually do in practice”) with a dual focus on the assessment of learning (i.e., summative feedback) and assessment for learning (i.e., formative feedback) [1–3]. As a result, direct observation of a trainee-patient encounter has become an increasingly prominent feature of assessment. Direct observation tools have been developed for general clinical skills (e.g., miniCEX) and for focused tasks, such as electromyography, teamwork, laparoscopy, ultrasound-guided anesthesia, handoff, and follow-up visits [4–11].

Implementation of workplace-based assessment has encountered significant challenges. One common barrier has been the lack of time and competing demands such as clinical workload that interfere with the ability of faculty to complete these assessments [12, 13]. In order to facilitate more efficient capture, delivery, and aggregation of assessment data, mobile applications have been developed and tested in multiple specialties (e.g., pediatrics, surgical specialties, internal medicine) and with multiple frameworks (milestones, competencies, and entrustment scales) [14–21]. A second important barrier has been challenges with the assessment frameworks; the competencies and milestones used on workplace-based assessments are viewed by some as too numerous, too granular, and/or too abstract for educators to use [22]. Entrustable professional activities (EPAs) have emerged as an assessment framework that translates competencies into clinical practice in a more holistic fashion compared with milestones [23]. Multiple specialties have developed and implemented EPAs [24–26].

Very little has been published on mobile apps that utilize the EPA framework to capture assessment data, though there are numerous initiatives underway. Most of the published apps utilize milestones or competencies as the assessment framework. For example, the surgical specialties have developed two related assessment approaches, the O‑SCORE and SIMPL. The O‑SCORE utilizes levels of supervision anchors (e.g., “I had to talk the trainee through …”) for each of nine components of any surgical procedure (e.g., case preparation, postoperative plan) with a final yes/no determination of whether the trainee is ready to perform the procedure independently [27]. It does not use a level of supervision scale for the overall activity (which is not necessarily an EPA). SIMPL is a mobile platform that incorporates three questions, one of which uses a level of supervision scale for the overall activity but the activities typically are not EPAs [18]. Finally, Warm et al. have published on large WBA datasets captured by mobile devices; this work employs “observable professional activities” (i.e., often tasks nested within an EPA) and has not focused to date on the mobile platform itself [28].

In an effort to bring together the EPA framework with smartphone technology, we designed and implemented a WBA that employs a mobile app to assess EPAs based on direct observation. An initial study indicated that the app generated high quality narrative feedback and entrustment scores that correlated with resident experience [29]. While this and other EPA apps are being implemented across numerous settings and specialties, we know little about implementation barriers and enablers. To date, most studies of assessment apps have examined apps that use frameworks other than EPAs and have focused on outcomes such as end-user satisfaction via surveys (e.g., attitudes), the quality of the feedback (e.g., specificity), and feasibility (e.g., time to complete) [15, 18–20, 30–36]. A few of these studies have identified barriers (e.g., competing demands on faculty time and lack of a physician champion) and enablers (perceived value) to implementation [15, 17, 19]. No study to date has used implementation science frameworks to focus on the implementation process itself.

If EPA apps and, more generally, smartphone-based applications, are to be successfully incorporated into programmatic assessment, a better understanding of implementation barriers and enablers will be necessary. To address this gap and improve subsequent implementation, this study explored the barriers and enablers of adoption of an EPA-based app. The study used the Consolidated Framework for Implementation Research (CFIR), a “meta-theoretical” framework that provides an overarching typology of implementation and is commonly used to assess implementation of evidence-based interventions in a variety of settings, including medical education [37–39].

## Methods

### Study design and ethics

This is a qualitative study that applied the CFIR methodology [37]. Because our focus was on identifying implementation barriers and enablers in order improve how other programs incorporate similar apps in the future, we chose a methodology from implementation science [12, 39]. The CFIR examines implementation across five major, interacting domains: intervention (e.g., perceptions about the relative advantages of the intervention), inner setting (e.g., the clinic in which the supervisory encounter between faculty and residents occurs), outer setting (e.g., department and hospital policies, priorities, incentives and culture), individual characteristics (e.g., knowledge and beliefs about the intervention, personal use of the app), and implementation process (e.g., strategies and tactics such as engaging appropriate stakeholders) [40].

Ethical approval was obtained from the Institutional Review Board at Northwell Health (IRB#: 19-0011).

#### Setting and participants

This study was conducted in a psychiatry resident outpatient continuity clinic of a large, academic teaching hospital. Residents spent one half day a week in the clinic during their second and third year of training with the same attending. Each week, the attending directly observed second year residents for two hours and third year residents for one hour as they conducted new patient evaluations and follow-up visits. Faculty had no other obligations during the clinic other than working with their assigned resident. Prior to the implementation of the EPA app, faculty used a paper-based direct observation tool that included a comprehensive 27-item checklist, an overall EPA rating, and prompts for both reinforcing and corrective comments. This tool had been studied in several settings with evidence for validity and generates, on average, five highly specific comments with a 3:2 ratio of reinforcing to corrective [11, 41–43]. All faculty agreed to participate. Two residents who were invited declined to participate.

### Intervention

Design features of the mobile app and the quality of the assessment data generated have been reported in a prior study [29]. In brief, we developed the app for the iOS platform in Xcode, Apple’s suite of software development tools. The app was written in the Swift programming language. Data were uploaded to Google’s Firebase cloud service, and emailed directly to residents via a Firebase Cloud Function. To make the interface as intuitive and hassle free as possible, we adhered closely to the iOS Human Interface Guidelines, a set of documents published by Apple, whose aim is to improve the user experience. Iterative refinements were made based on field testing performed by the study authors and one other faculty member. A pilot study of the EPA app determined that faculty required 70 seconds to complete an assessment and each assessment generated, on average, a single, behaviorally specific, high quality corrective comment [29].

The EPA app was implemented in August 2017. Faculty were asked to use the mobile app to complete one evaluation during each continuity clinic in which the resident saw at least one patient. After observing the patient encounter, the app required faculty to select the relevant EPA—in this context either a “diagnostic interview” or a “medication management visit”—and then complete a two-part assessment: 1) assign a level of supervision that the resident requires based on the single observation; and 2) provide a comment in response to the prompt “one thing the trainee can do to advance to the next level”. Once the faculty completed the assessment, a copy was immediately emailed to the resident and faculty member.

Approximately once a month, use of the app was monitored by study authors via a dashboard that summarized the number of completed assessments for each dyad. When the dyad had been inactive during the prior time interval an email reminder was sent to the faculty, encouraging them to continue using the app. When a dyad was inactive for several time periods, the study authors reached out to the faculty member to see if they needed help with the app. Faculty development consisted of written instructions and a 30-minute one-on-one meeting to install and practice using the app. In addition all faculty attended three 1‑hour trainings prior to the start of the intervention on direct observation, EPA-based assessment, and narrative feedback, respectively. Residents also received a single 30 minute orientation to the EPA app and the expectations for its use.

### Interview content

Separate interview guides were created for faculty and for residents. CFIR contains 26 constructs across the five domains. Not all constructs are relevant to a given context. Sample interview questions for each construct are available on http://cfirguide.org. The research team selected constructs and questions relevant to the EPA app implementation from four of the domains: intervention characteristics, individual characteristics, inner setting, and outer setting. The constructs from the process domain apply primarily to those who are responsible for the planning and execution of the program, i.e., the residency program and clinic leadership. Because our focus was on the faculty and resident experience, we did not interview the EPA app implementation leaders and therefore did not include any of the process domain constructs in the guide. The questions were then tailored to gather specific information relevant to how faculty and residents experienced the app. Following a pilot interview with a faculty member and with a resident, minor changes were made for clarity.

### Data collection

Structured interviews were conducted by a study author (RS) from February to March 2019. We invited faculty members and residents in the intervention clinic to participate. Written informed consent was obtained from each participant. The study author explored participants’ reactions to each question. Each interview was audiotaped, transcribed, and de-identified.

### Data analysis

Anonymized transcripts were uploaded to Dedoose (version 8.2.14 for Windows) for data analysis and management. Two authors (RS, JS) conducted directed content analysis, a deductive process that applies an existing theory or framework to guide initial coding [44, 45]. Transcripts were independently coded in iterations of two. Each transcript was segmented into excerpts, i.e., a linguistic unit (e.g., sentence or paragraph) that expressed a single idea. Each excerpt was then deductively assigned to one of the four CFIR domains used in this study (i.e., intervention characteristics, individual characteristics, inner setting, and outer setting). The same two authors then inductively assigned codes (e.g., technical interface or competing demands) to each excerpt. After independently coding each batch of two transcripts, two authors (RS, JS) compared how they segmented the data into excerpts and the CFIR domain and codes they assigned to each excerpt. Although interview guide questions were organized by CFIR domain, we coded excerpts independently of the domain under which the question was categorized in the interview guide. Differences were discussed with the lead author (JQY) until consensus was reached both with respect to the excerpt segmentation, the assigned CFIR domain, the coding scheme itself and the assignment of codes to a given excerpt. In subsequent meetings, all study authors examined the codes within each CFIR, identified relationships between the codes, combined codes into categories and then constructed themes relevant to each domain. After eight faculty and 10 resident interviews, three authors (RS, JS, JQY) all perceived that conceptual sufficiency had been reached, i.e., the codes appeared to capture the essence of the phenomenon without requiring further modification [46].

The first author was also the program director for the psychiatry residency in which this intervention took place. Participants were informed of the first author’s role. Several steps were taken to ensure that faculty and residents participated voluntarily and openly. The first author did not participate in recruitment, interviews, or initial coding. He only participated in coding when the two primary coders disagreed and only viewed excerpts that had been de-identified.

### Reflexivity

The study authors are all engaged in assessment in graduate medical education and have experienced the challenges of gathering WBA data via paper forms. We anticipated that smartphone-based apps for competency-based assessment may be a much easier interface, especially for faculty. This positive bias could have influenced analysis. We also expected that the smartphone-based platform may result in fewer and perhaps lower quality comments compared with the paper forms we had used in the past. This assumption had the potential to provide a negative bias to the analysis. To manage the influence of these assumptions on our analysis, study team members were asked to routinely reflect on their assumptions and to verbalize how they may be affecting the process of creating codes and themes.

## Results

Below, we describe the major themes within each CFIR domain and provide exemplar quotes to illustrate how the themes were communicated. The perceived advantages and disadvantages of the EPA app compared with the pre-existing paper-based assessment tool are described in each relevant CFIR domain.

### CFIR domain—Intervention characteristics

This refers to how participants perceived the quality of the app, including design and ease/difficulty of use, and positive and negative effects of the app.

#### Ease of use:

Faculty felt the app was easy to use and intuitive, from initial setup to routine use. Faculty experienced few, if any, bugs or technical problems. One faculty commented: *“I’m not very good with the phone … I found [EPA app] easy to use. I had no issues with it.” (Faculty_3) *No faculty member cited an aspect of the app that was confusing or frustrating. Similar to faculty, residents experienced no technical challenges. Faculty and residents preferred the electronic format over paper. For faculty, the paper-based forms required remembering to bring the form to an observation and to then submit once completed while for residents they cited the hassles of storing and retrieving the completed paper forms.

#### Feedback timeliness and frequency:

All faculty and residents reported that the EPA app facilitated timely and frequent feedback. Faculty attributed this impact to the quickness and ease with which an assessment could be completed:* “[EPA app] made giving feedback still formal and objective, but also quicker, so you could spend more time interacting with the resident and doing more verbal feedback … it allowed for more face-to-face feedback …” (Faculty_7)* With regards to ease of use and time to complete, faculty much preferred the EPA app to the longer, paper-based assessment tool: *“I think [EPA app] is much more user friendly and much more likely to be completed and much more efficient [than paper forms] …” (Faculty_2)* Similarly, residents appreciated receiving the written (electronic) feedback within minutes of the patient encounter:* “After my supervisor gave me real-time verbal feedback … probably 15 minutes after I left I would get an email with feedback.” (Resident_3).*

#### Feedback quality:

Most faculty appreciated that the app prompted only for corrective (and not reinforcing) narrative feedback. In particular, faculty described how the corrective prompt served as a “forcing function” to do the hard work of constructing such feedback:* “It’s helpful because every interaction there’s usually at least one area for improvement and this forces you to identify that.” (Faculty_5)* A few faculty felt discomfort with not also having a prompt for reinforcing feedback; they worried that their feedback may be misperceived as discouraging or unsupportive.

All faculty described how the act of constructing written feedback within a smartphone-based app resulted in much briefer feedback compared with the paper-based tools where they might write multiple feedback points, each one with considerably more narrative. Faculty perceived this design feature to be beneficial as it required them to distil their feedback into a single, brief point. Some faculty thought a single, brief point may even be more beneficial than several, longer points.

*“People are more likely to listen to it or pay attention to it when it’s something short and digestible, so that’s what I try to do. It forced me to do that too, which was good for me.” (Faculty_1)*

*“[I like] the fact that you have to provide very succinct feedback [for the EPA app] and it always makes me think about the most helpful piece of feedback for the resident to take away.” (Faculty_6)*

Most residents expressed appreciation for the brief “take home” messages facilitated by the EPA app. For example, one resident commented: *“I think that it’s helpful to get one main point for organized feedback … I like to say ‘That’s one point I can take away from this session today.’” (Resident_4).*

However, for both faculty and residents, there were drawbacks to the concise, succinct, and easy to digest characteristics of the EPA app’s written feedback. Both groups believed that the EPA app, compared with the longer paper-based checklist with prompts for both reinforcing and corrective comments, generated feedback that was less nuanced, comprehensive, and balanced. One resident said: *“I guess the app feedback is less detailed and that made it not as helpful.” (Resident_3) *Another commented: *“I’m not sure the app asks for something positive for the comments, which means in some senses the feedback’s a little less thorough.” (Resident_2) *Similarly, a faculty member noted*: “I think the longer, paper-based form is more effective, because it’s more comprehensive.” (Faculty_7).*

Both faculty and residents resolved these tradeoffs with the notion that an ideal assessment program would include both types of assessments, as captured by these excerpts:

*“I think something would be lost if only one was used to the exclusion of the other. I think it might be an ideal mix of primarily using the phone because of its ease of use and it’s the easy way of generating a lot of data, but then periodically doing the paper one because it reminds us of some trees, not just the forest.” (Faculty_1)*

*“I think the PSCO (paper-based form) is more thorough. The PSCO’s probably more accurate, encapsulates the experience more accurately. So, that’s the advantage of the PSCO. The disadvantage of the PSCO is that it’s a little more time-consuming to do. I think the advantage of the app is that it’s very quick. The disadvantage is that it’s not detail oriented, so I think it would probably be better for a large, summative block of feedback as opposed to looking at an individual visit.” (Faculty_5)*

*“I feel like I think it’s that same trade-off. Like the PSCO is more detailed and more specific, but it runs the risk of just having attendings just check-off a ton of boxes without giving it a lot of thought because there’s too much stuff to do. Whereas the app has less detail, and it’s less broad, and has less information, but there’s only one thing to do, so that one thing ends up being very specific and usually quite helpful.” (Resident_3)*

Some faculty wondered if the more comprehensive paper-based tool might be especially preferable early in a resident’s training or faculty’s teaching when both may benefit from the checklist which makes explicit the standards of competency and may facilitate more specific feedback.

#### Frame of reference:

All faculty described the orientation training as sufficient. Yet, most faculty expressed not understanding adequately the entrustment scale or EPA framework*: “There are buttons that explain what the definitions are. But it actually doesn’t explain if it’s asking for what happened versus what you’re recommending.” (Faculty_2).*

### CFIR domain—Characteristic of individuals

This refers to how the user’s own beliefs or characteristics may affect the intervention. These characteristics include how they use the app in practice and their confidence in doing so.

Faculty used the app shortly after providing verbal feedback to the resident, either in the presence of the resident, or shortly after they left, typically no more than 20 minutes after observing the encounter.

#### Confidence and excitement:

Most faculty and all residents had a positive emotional reaction to using the app. Faculty had a high level of confidence in using the app, regardless of their general level of confidence with technology*: “Since I’ve had smartphones and have been using apps for a bunch of years now, I’m fairly comfortable with new technology in general.” (Faculty_7) *Residents expressed a similar positive disposition to use of an electronic rather than paper format:* “I come from a generation where everything is done electronically … it’s easier for us to access that because that’s what I grew up with.” (Resident_6).*

#### Use of EPA app in presence of patients:

Some faculty expressed discomfort with the use of the EPA app during a patient visit. These faculty worried that patients would perceive use of the EPA app as lack of interest, not paying attention, or even rude: *“I found it very weird being on my phone with a patient in the room. Computer’s one thing because it’s the electronic medical record … but the phone just feels rude because people don’t know what I’m looking at.” (Faculty_4)* Instead, most faculty took brief notes on paper during the patient encounter so they could remember key points and examples when providing the verbal and written feedback. In this respect, faculty found the pre-existing paper-based tool more seamless. Residents did not comment on this.

#### Resident engagement with the feedback:

All residents appreciated receiving the written feedback electronically soon after the patient encounter. While some faculty assumed that residents would return to the feedback in order to see how they were progressing, almost all of the residents indicated that they never looked at the emailed feedback after the initial view.* “I may be looked at it for like a second, however long it takes to read a sentence … then I probably would have deleted it.” (Resident_8).*

### CFIR domain—Inner setting

In this study, the inner setting referred to the ease of implementation within the clinic itself.

#### Clinical demands:

Most faculty identified clinical demands as the main barrier to implementation. Faculty had no clinical obligations of their own when precepting the residents; this feature of the program facilitated the direct observation and assessment activities. Yet, faculty still experienced interruptions. And, more significantly, clinical demands from the resident’s panel could impact engagement with the EPA app (e.g., a patient with a brief appointment presents with unexpected acuity leading to a backlog in the resident’s clinic). When faculty experienced competing demands, they prioritized verbal feedback over completing the assessment in the app. These perceptions are represented well by the following two excerpts:

*“[EPA app is] another thing to do. When it gets busy and I’ve already given verbal feedback, sometimes it falls by the wayside.” (Faculty_4)*

*“Usually we’ll [use the EPA app] if time permits. If we only have one patient scheduled in the next hour or so [my supervisor says] let’s do an app.” (Resident_4)*

### CFIR domain—Outer setting

In this study, the outer setting refers to how the app did or did not meet the needs of the hospital’s clinical enterprise and the department’s educational program.

#### Organizational values:

A majority of faculty and residents felt that the app was a good fit with the values and norms of the organization, as well as their own values and norms. Faculty and residents perceive the organization as prioritizing innovation in clinical and educational practice. A faculty member commented: *“I think [EPA app] does fit with the value of being a forward-thinking, progressive kind of educational environment …” (Faculty_3) *Similarly, a resident observed:* “I think it fits well. We value high quality education, learning, research, making changes in residency.” (Resident_8) *Many cited that the department had established a clear expectation that supervising faculty should use the mobile app or other assessment instruments. Moreover, faculty and residents described how the EPA app aligned especially with the department’s visible efforts to use digital technology to improve access to high quality care (e.g., smartphone-based cognitive behavioral therapy).

## Discussion

This study identified enablers and barriers to engagement with the EPA app that have implications for future iterations of this and other EPA apps (Tab. 1). A number of enabling factors were identified. Both faculty and residents found the app easy to use, glitch free and efficient in facilitating feedback soon after the observed patient encounter. None of the participants experienced the EPA app as burdensome or difficult to navigate, a common complaint of online WBA tools [47, 48]. Faculty appreciated how the EPA app forced them to distill their feedback into a single point. Both faculty and residents expressed positive affective reactions. These important enabling factors highlight the critical importance of the design process used in developing an assessment app. The design of the EPA app followed user-interface guidelines and prioritized a simple and efficient user interface which meant accepting certain compromises such as not collecting information about the patient complexity, only having a single text box for corrective comments, and placing detailed anchor language in information buttons to reduce the text on the main screens. The design process also incorporated revisions based on feedback from testing sessions with several faculty members.

**Table 1:** Facilitators and barriers to engagement with the EPA app

CFIR domain	Facilitators	Barriers
*Intervention characteristics*	– Sufficient training prior to use – Few, if any, technical challenges – EPA app intuitive and easy to use, especially compared with paper-based assessment tools – Feedback timely and frequent – Feedback quality high—behaviorally specific and salient – User interface forced succinct feedback with a single take home message for the resident	– Residents and faculty see the value of assessment tools (such as the paper-based form also used in the clinic) which generate more comments that are more detailed, nuanced, and comprehensive – The absence of a checklist, while making the app easier to use, led to less systematic observation and feedback – No reinforcing comments – Most faculty did not understand the entrustment scale and/or the EPA framework – Faculty prefer paper-forms for discretely jotting down feedback points while observing
*Characteristics of individuals*	– Excitement about the use of app-based technology – High confidence in use of the app – Faculty appreciated how the interface forced synthesis and distillation of their observations into a single, concise feedback point	– Faculty worry that use of the EPA app during patient encounters may convey lack of respect and attention – Residents reviewed emailed feedback briefly, then rarely referred to it again – Faculty prioritized verbal feedback over app completion when short on time
*Inner setting*	– Faculty time protected for the sole purpose of directly observing the resident and giving feedback – Monitoring of app utilization by the program	– Clinical demands, especially from the residents’ panels of patients, often resulted in the EPA app assessment not being completed
*Outer setting*	– The app aligned with the organization’s emphasis on innovation—especially regarding the use of measurement and technology—in clinical and educational practice	

In addition, the protected time for faculty to observe and complete the assessments without clinical obligations of their own was crucial. The barrier of competing demands has been consistently reported in studies of other workplace-based assessment strategies [12, 13, 15, 19]. These findings have led many to advocate for models that provide faculty with dedicated (compensated) time for direct observation and feedback [12, 13, 49]. This recommendation, as implemented in our study, facilitated engagement with the EPA app.

Moreover, faculty and residents perceived the app as aligned with the hospital’s and department’s philosophy which illustrates the importance of linking any EPA app implementation to the underlying values and sources of pride in an organization.

However, several important barriers to engagement were identified. Most faculty expressed inadequate understanding of the scale or framework. Prior research has identified inadequate understanding of the performance dimensions and frame of reference as dominant problems in WBA programs [13]. While proponents of the EPA framework contend that EPAs are intuitive for clinical faculty compared with the milestones framework, the faculty in this study applied the EPA framework inconsistently. Despite expressing satisfaction with the training, faculty evidently required more training and support over and above the 30 minute orientation to the app and the three one-hour trainings which covered EPAs and entrustment scales [50]. This is consistent with the general finding in WBA research that repeated trainings are necessary to establish and maintain a shared mental model among raters [51, 52]. This may serve as a caution to not under-estimate the effort and time it takes for faculty to learn how to use the EPA framework. Even if the EPA framework is easier for clinical faculty to initially grasp, how faculty use the framework in practice may be problematic.

In addition, some faculty did not use the EPA app as frequently as intended due to clinical demands that took precedence. In the setting of our study, it is somewhat surprising that this barrier persisted, though perhaps to a lesser extent than reported in other studies, given how seemingly “little” time the EPA app takes (70 seconds on average), how high the enthusiasm for the app was, and that faculty had no other obligations when supervising their resident in the continuity clinic. Even with the faculty time protected, faculty still were interrupted with concerns related to their panel of patients. Moreover, demands of patients from the residents’ own panels also disrupted the direct observation and feedback. For example, if a resident were fully booked and then had an unscheduled acute patient present, the faculty and resident would dispense with the feedback. Neither of these disruptions were anticipated. This highlights how even with faculty protected time, faculty may still encounter significant interruptions and, in addition, steps (e.g., longer appointment times or blocked off appointment slots) must be taken to establish buffers within the residents’ clinics against unexpected demands that might interfere with feedback conversations and app completion.

Moreover, residents report that they rarely, if ever, referred to the emailed feedback after an initial brief review. This contrasts with the faculty expectation that the emailed feedback would be revisited at future time points. This finding is concerning and represents a significant threat to the impact on learning and, ultimately, the validity of a competency-based assessment program such as the EPA app [49]. Providing feedback, even if purely formative, is not enough to stimulate growth. Learners must review, reflect, discuss, and apply the feedback [49, 53–55]. Yet, medical students and residents typically are not self-regulated learners who engage in reflection and self-improvement on their own accord, a finding seen in both formative and summative assessment [56, 57]. Two interventions seem relevant. Aggregating and visualizing the performance data onto a dashboard may help trainees perceive trends and more easily find value in re-visiting feedback they have received over time [54]. In addition and more important, residents may need longitudinal coaches that create a safe place in which they learn how to identify growth edges and set action plans [49, 54].

Finally, while faculty and residents appreciated the concise, single-point feedback facilitated by the EPA app, both also noted the value of the pre-existing systematic, paper-based tool that generated more comprehensive, balanced, and nuanced feedback. This outcome stemmed from the intentional design decision to limit the assessment to a single rating and a single text box in order to maximize efficiency and ease of use. Longer direct observation tools have been shown to generate multiple comments per observation [43, 48, 58]. We do not know what the optimal number of comments is from a learning and behavior change perspective, but this finding suggests that an overall program of workplace-based assessment may want to include a mix of assessment tools that generate both brief and more detailed comments. Moreover, this finding raises several questions about the design of the EPA app, such as whether a comprehensive checklist and/or a second comment field that prompts for reinforcing feedback should be added. We would recommend adding the second narrative field but are reluctant to add a checklist, especially a 27-item one, which would make completion of an assessment much more burdensome, especially on the smaller screen of a smartphone.

Limitations of this study include a small sample size from a single outpatient clinic at a single institution. Implementation barriers and enablers are inevitably related to local contextual factors. The lessons from this study may not be generalizable. At the same time, many of the findings are congruent with studies of other types of mobile apps which makes us more confident in our findings. In addition, the interview questions and coding processes were shaped by a specific theoretical framework that may not have captured important dimensions of the EPA app experience. However, we believe this approach was appropriate given our focus on the implementation enablers and barriers.

## Conclusion

In summary, this qualitative study using the CFIR framework identified key enablers and barriers to faculty and resident engagement with the EPA app. The findings support ease of use and utility but also highlight important barriers such as competing demands, variable faculty understanding of the assessment framework, lack of resident use of the feedback beyond initial receipt, and salient tradeoffs when comparing comments generated by the app versus longer, more detailed paper-forms. Educators should utilize app development guidelines that optimize the user interface. Future research and implementation efforts should especially focus on how best to train faculty and to catalyze residents to engage in ongoing review and reflection with the support of a coach.

## Caption Electronic Supplementary Material

Faculty Version Interview Guide

Resident Version Interview Guide
