# Supplementary material for: A mobile app to capture EPA assessment data: Utilizing the consolidated framework for implementation research to identify enablers and barriers to engagement
Source: Perspect Med Educ. 2020 Jun 5;9(4):210–9. doi: 10.1007/s40037-020-00587-z (PMC7459074; doi:10.1007/s40037-020-00587-z)
Supplement: Supplementary file 1 — Faculty Version Interview Guide [file 40037_2020_587_MOESM1_ESM.docx]

**Introduction**

- Thank you for agreeing to participate in this interview regarding the direct observation assessment tools you used in the continuity clinic over the past year. We are grateful for taking the time to talk with us. We will be audio-taping this interview so we have a full and accurate record of your thoughts.

**Intervention Characteristics (App)**

First we’ll talk about the characteristics of the EPA Feedback app.

**Demonstration**

- Please show me the App and how you submit an evaluation?
- When did you start using the app? (What date?)

**Design Quality**

- Can you summarize the overall quality of the App?
- What, if any, bugs or technical problems did you have with the App?

**Complexity (& Ease of Use)**

I’d like to ask you to assess each part of the app.

- How easy or complicated was:
  - The initial set up on your phone?
  - To open up the app whenever you decided to use it
  - To select the resident and the EPA
  - To use the level of supervision scale
  - To type or dictate the feedback
  - How often did you refer to the ‘i’ buttons that provided explanatory information? In what ways was this helpful?
- Were there any other aspects of the app that were confusing or frustrating?
- How did you feel about the requirement to provide narrative feedback for each assessment?
- How did you feel about the length of the text feedback you provided in the App?
- How did you feel about the suggestion that the narrative feedback focus on how to advance to the next level of supervision?
- How did you feel about feedback being emailed directly to the learner?

**Adaptability**

- Are there any changes or improvements that you would like to see in the App?
- What components of the App were crucial/necessary and should not be altered?

**Characteristics of Individuals (App)**

Now I’d like you to walk me through how you used the app.

**Personal use of the app**

- Describe how you used the app.
- Did you start the assessment during the patient encounter?
- Did you take notes during the encounter?
- How soon after the observation did you press submit?
  - In the presence of the resident?
- Typically, when would you provide verbal feedback to the resident - before, during, or after? If after – how soon after?

**Knowledge & Beliefs about the Intervention**

- How effective was the App in your clinical supervision?
  - What aspects of the App most strongly enabled its use for clinical supervision?
  - What aspects of the App interfered with its use for clinical supervision?
- How did you feel about the App being used in your setting?
  - Did you have any feelings of anticipation? Stress? Enthusiasm? Why?

**Self-efficacy**

- How confident did you feel in using the app for clinical supervision?
  - What gave you that level of confidence (or lack of confidence)?
  - How long did it take to build that confidence?

**Other Personal Attributes**

- How comfortable do you feel with technology in general and how did this influence your use of the App?

**Outer Setting (App)**

Now I’m going to ask about how the app meets the needs of the resident.

**Learner Needs & Resources**

- How well do you think the App met the needs of the resident?
- In what ways did the App meet their needs?
  - *Probes if needed:*
    - *Faster feedback*
    - *More frequent feedback*
    - *More specific or constructive feedback*
- In what ways might the App have not helped or even hindered meeting the needs of the resident?
  - *Probes if needed:*
    - *Felt criticized*
    - *Felt self-conscious*
    - *Undermined credibility with the patient*
    - *Took time away from other activities such as discussing the treatment plan, informal discussion, more time with patient.*

**Inner Setting (App)**

- Now I’m going to ask about how the app fits into this organization.

**Tension for Change**

- Is there a need for this App in the residency program?
  - Why or why not?
  - How strong is that need?

**Compatibility**

- How well does the App fit with your understanding of the organization’s values and norms?

(*Can prompt with values relating to interacting with residents, e.g. purely verbal feedback versus documented on paper/electronically?)*

- How well does the App fit with your values and norms?

**Relative Priority**

- How did the App fit in with other supervision or clinical activities?
  - Did the App ever take a backseat to other activities? Why? What other activities?
  - How important did you think it was to use the App compared to the other priorities?
  - Were there competing priorities that got in the way of using the App?
  - Did using the App mean other important activities were not done?

**Organizational Incentives & Rewards**

- Were there any incentives to help ensure that the App was successful?
  - What was your motivation for wanting to help ensure the App was successful?

**Goals & Feedback**

- Did the creators of the App set goals related to its use?
  - *(If yes)* What are the goals? Did you feel that you met them?

**Access to Knowledge & Information**

- What kind of training did you receive prior to using the App?
  - Did you feel the training was sufficient?
  - What was missing from the training?
