# Supplementary material for: A mobile app to capture EPA assessment data: Utilizing the consolidated framework for implementation research to identify enablers and barriers to engagement
Source: Perspect Med Educ. 2020 Jun 5;9(4):210–9. doi: 10.1007/s40037-020-00587-z (PMC7459074; doi:10.1007/s40037-020-00587-z)
Supplement: Supplementary file 2 — Resident Version Interview Guide [file 40037_2020_587_MOESM2_ESM.docx]

**Introduction**

- Thank you for agreeing to participate in this interview regarding the direct observation assessment tools you used in the continuity clinic over the past year. We are grateful for taking the time to talk with us. We will be audio-taping this interview so we have a full and accurate record of your thoughts.

**Intervention Characteristics (App)**

First we’ll talk about the characteristics of the EPA Feedback app.

**Design Quality**

- Can you summarize the overall quality of the emailed feedback?
- Were you aware of any technical problems or bugs with the app or emailed feedback?

**Complexity (& Ease of Use)**

I’d like to ask you to assess each part of the emailed feedback.

- How easy or complicated was:
- Interacting with the email
  - Accessing the email? (phone, desktop etc…)
  - Opening up the email
  - Reading the email
- Remembering the context in which the email was generated
  - Patient visit
  - Attending
  - Post-visit conversation with the attending
- Identifying the key feedback in the email:
  - Supervision level
  - Feedback
- Were there any aspects of the app that were confusing or frustrating?
- Was receiving feedback via the email disruptive in any way?

**Relative Advantages**

In many settings, we do not use any feedback tool like the iPhone app or the P-SCO. In those situations, the feedback comes only from the verbal interactions with the attending. I’d like to ask you to assess the advantages of receiving feedback via the app versus no feedback tool.

- In what ways is having feedback come from the app in addition toverbal interactions better?
- In what ways is it worse?

**Adaptability**

- Are there any changes or improvements that you would like to see in the emailed feedback?
- What components of the emailed feedback were crucial/necessary and should not be altered?

**Characteristics of Individuals (App)**

Now I’d like you to walk me through how you personally used the emailed feedback.

**Personal use of the app/email**

- How did the supervisor use the app?
  - Describe how your supervisor used the app in supervision.
  - Did your supervisor start completing the app during the patient encounter?
  - How soon after the observation did you received the emailed feedback?
    - Did your supervisor complete the app in your presence? If so – how was that for you?
  - Typically, when did your supervisor provide verbal feedback to you in relation to the emailed feedback? - before, during, or after? If after – how soon after?
- Tell me how you used the emailed feedback?
  - Typically, when did you look at the emailed feedback, and how much time did you spend looking at it?
  - What did you do with the email? *(e.g. archived, deleted, ignored, etc)*
  - Did you ever find yourself looking at or reflecting on an old emailed feedback?
  - Did you have any feelings of anticipation? Stress? Enthusiasm? Why?

**Knowledge & Beliefs about the Intervention**

- Stepping back, what do you think about the emphasis or value being placed on this app as a way to give feedback?
- What do you think about the level of supervision scale?
- How did you feel about the narrative feedback focusing on one thing you could do to advanceto the next supervision level?

**Self-efficacy**

- How confident do you feel in your ability to understand the feedback and what it is really getting at?
- How confident do you feel in your ability to use the feedback to improve or grow?

**Outer Setting (App)**

Now I’m going to ask about how the emailed feedback meets your needs as a learner.

**Learner Needs & Resources**

- How well do you think the App met your needs as a learner?
- In what ways did the emailed feedback meet your needs?
  - *Probes if needed:*
    - *Faster feedback*
    - *More frequent feedback*
    - *More specific or constructive feedback*
- In what ways did the emailed feedback not help or even hinder meeting your needs as a learner?
  - *Probes if needed:*
    - *Felt criticized*
    - *Felt self-conscious*
    - *Undermined credibility with the patient*
    - *Took time away from other activities such as discussing the treatment plan, informal discussion, more time with patient.*
    - *Distracted the attending*
- How relevant/helpful was the feedback that was emailed to you from the app?
  - Supervision level
  - Narrative feedback
- How did you feel about the length/level of detail of the feedback in the email?

**Inner Setting (App)**

Now I’m going to ask about how the EPA App fits into this organization.

**Tension for Change**

- Is there a need for this App in the residency program?
  - Why or why not?
  - How strong is that need?

**Goals & Feedback**

- What is your understanding why feedback via the app was introduced?
  - What are the goals?
  - Did you feel that you met them?

**Compatibility**

- How well does the App fit with your understanding of the organization’s values and norms? (*Can prompt with values relating to interacting with residents, e.g. purely verbal feedback versus documented on paper/electronically?)*
- How well does the App fit with your values and norms?

**Relative Priority**

- How did the App fit in with other supervision or clinical activities?
  - When did the App take a backseat to other activities? Why? What other activities?
  - How important did you think it was to use the App compared to the other priorities? *(e.g. verbal feedback, discussion, logistical tasks, etc).*
  - Did using the App mean other important activities were not done?

**Access to Knowledge & Information**

- What kind of training did you receive prior to using the App?
  - Did you feel the training was sufficient?
  - What was missing from the training?
